# Competence Mining of Vancomycin (VAN) in the Management of Infections Due to Bacterial Strains With High VAN Minimum Inhibitory Concentrations (MICs): A Novel Dosing Strategy Based on Pharmacokinetic/Pharmacodynamic Modeling

**DOI:** 10.3389/fmicb.2021.649757

**Published:** 2021-04-22

**Authors:** Xiangqing Song, Meizi Zeng, Yi Wu, Yong Pan

**Affiliations:** Department of Pharmacy, Hunan Cancer Hospital, The Affiliated Cancer Hospital of Xiangya School of Medicine, Central South University, Changsha, China

**Keywords:** vancomycin, methicillin-resistant **Staphylococcus aureus**, MRSA, **Enterococcus**, pharmacokinetic/pharmacodynamic, Monte Carlo simulation

## Abstract

The increasing emergence of bacterial strains with high VAN MICs (BS_*H*__–__*V*__*AN–*__*M*_), such as *Enterococcus faecalis*, *Enterococcus faecium*, *Staphylococcus aureus*, *Staphylococcus epidermidis*, and *Streptococcus bovis*, results in growing concern that VAN is not effective against these isolates. Due to the limited data on VAN against BS_*H–VAN–M*_ and the application limits of drugs currently considered to be effective for BS_*H–VAN–M*_, exploration of “new usages for old drugs” is reasonable to improve and maximize the efficacy of existing antibiotics. This study aimed to construct a novel dosing strategy to mine the competence of VAN in the management of BS_*H–VAN–M*_ infections. Herein, we optimized the traditional intermittent i.v. infusion (TIII) method to create an optimal two-step infusion (OTSI). With pharmacokinetic (PK)/pharmacodynamic (PD) modeling at the targeted ratio of the daily area under the concentration-time curve (AUC_0__–__24_) to the minimum inhibitory concentration (MIC) (AUC_0__–__24_/MIC) of 400, we used Monte Carlo simulations to evaluate the efficacy of 25 VAN regimens (including 15 OTSI regimens and 10 TIII regimens with daily doses of up to 6 g) to treat pneumonia, meningitis, sternal osteomyelitis, mastitis, pleuritis, bacteremia, and bacterial pericarditis resulting from isolates with MICs of ≤64 mg/L and to the current *E. faecalis*, *E. faecium*, *S. aureus*, *S. epidermidis*, and *S. bovis* populations with a pooled MIC distribution. Our data indicated that 4 g/day VAN, with an OTSI but not a TIII, for mastitis, pleuritis, bacteremia, and bacterial pericarditis due to isolates with MICs of ≤4 mg/L or to the current *E. faecalis*, *S. aureus*, *S. epidermidis*, and *S. bovis* populations achieved the desired PK/PD exposure at the AUC_0__–__24_/MIC target of 400. This study suggests the superiority and feasibility of OTSI relative to TIII for the competence mining of VAN against BS_*H–VAN–M*_ from the perspective of PK/PD and provides a new resource for understanding how PK/PD modeling shapes the performance of VAN to meet the growing challenges of BS_*H–VAN–M*_ infections.

## Introduction

The increasing emergence of bacterial strains with high VAN MICs (BS_*H–VAN–M*_), such as *Staphylococcus aureus*, methicillin-resistant *Staphylococcus aureus* (MRSA), and *Enterococcus faecium* (The Micron Group, 2018; European Committee on Antimicrobial Susceptibility Testing (EUCAST), 2020), which are defined as any isolate displaying VAN MICs of ≥2 mg/L in the broth microdilution or of ≥1.5 mg/L in the *E*-test (Song et al., 2017), has posed significant public health threats and must be urgently addressed according to the antimicrobial resistance data released by the World Health Organization (WHO, 2017; Tacconelli et al., 2018). Infections due to these isolates not only cause growing concern that VAN is becoming disadvantageous (Sakoulas et al., 2004; Mavros et al., 2012) but also have severely limited treatment options.

Currently, some traditional drugs (e.g., daptomycin, linezolid, quinupristin-dalfopristin, tetracyclines, and chloramphenicol) and several newly approved agents (e.g., tedizolid, telavancin, oritavancin, and dalbavancin) for VAN-resistant *Enterococcus* (Barber et al., 2015), together with ceftaroline and tigecycline for MRSA (Gould, 2013), in monotherapy exhibit good activity. Unfortunately, however, they are limited due to either their significant shortcomings (Barber et al., 2015) [e.g., treatment-emergent side effects and drug interactions for daptomycin and linezolid (notably daptomycin) despite being well studied, higher morbidity and mortality and less tolerability for quinupristin-dalfopristin, and lack of sufficient documentation for tetracyclines and chloramphenicol] or the lack of sufficient clinical data. Worse, these drugs considered to be effective for BS_*H–VAN–M*_ are still not available even in tertiary hospitals in some regions (e.g., our hospital). Likewise, combination therapies of daptomycin or VAN with a β-lactam, or daptomycin with trimethoprim-sulfamethoxazole, or linezolid with daptomycin (or VAN or gentamicin or fosfomycin), which are considered synergistic for BS_*H–VAN–M*_ and may eventually prove to be more effective than monotherapy, particularly in “salvage” situations (Barber et al., 2015; Holubar et al., 2016; Chen et al., 2020), may also be limited since (1) the synergy of these combinations were derived mainly from retrospective studies or *in vitro* model, resulting in a lack of evidence of prospective randomized trials for a definitive answer on the synergy; and importantly (2) unavailability of daptomycin and linezolid in some hospitals is still an important factor that prevents this form of therapy. Antimicrobials, even novel and effective drugs, with a difficult balance of safety, efficacy, availability and other potential issues (e.g., cost and medical insurance protest), have often forced clinicians to rely on alternative options. Consequently, uncertainties remain for selecting the optimal treatment for BS_*H–VAN–M*_ infections.

If the dilemma without better options in the presence of BS_*H–VAN–M*_ infections can be broken through by optimizing the administration of the available drugs which have a currently reduced competency for such infections, it must be helpful for clinicians to treat such infections. Surprisingly, studies have shown that meropenem, with administration optimization, can still achieve sufficient PK/PD exposure against highly resistant bacterial isolates (Song et al., 2019), which has aroused substantial interest in exploring the “new usage for old drugs” to improve and maximize the performance of VAN against BS_*H–VAN–M*_ infections. If successful, this exploration will have important significance in clinic, especially considering the rapid progress against the MIC creep phenomenon, delays in the development of new alternatives, and lack of better treatment options. Due to the lack of outcome data, the updated guidelines regarding VAN therapy issued in 2020 by the American Society of Health-System Pharmacists (called the 2020 VAN guidelines) (Rybak et al., 2020b) encourage investigators to ascertain whether VAN can provide sufficient PK/PD exposure for the isolates despite their having an MIC susceptibility breakpoint as low as 2 mg/L. Given that positive studies on these isolates have currently rarely been performed and few attempts have been made to mine the competence of VAN against the increasing number of BS_*H–VAN–M*_ with MICs of ≥2 mg/L, this study aimed to construct a novel dosing strategy based on PK/PD modeling to exploit the maximum potentialities of VAN in the management of infections, especially for those resulting from isolates with MICs of ≥2 mg/L. We hope that our method will resolve the disadvantages of VAN in the growing presence of MIC creeping, especially in the absence of better alternatives.

## Materials and Methods

### Design of a Novel Dosing Strategy

**FIGURE 1:**
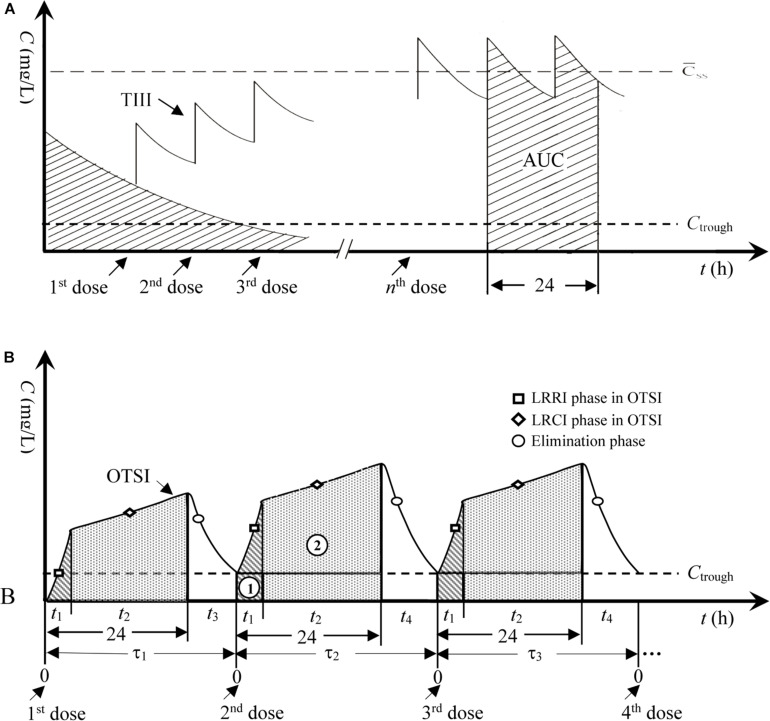
Concentration-time profiles for TIII and OTSI. **(A)** TIII, traditional intermittent i.v. infusion; AUC, area under the concentration-time curve; C̄_*ss*_, mean steady-state plasma concentration. **(B)** OTSI, optimal two-step infusion; τ_1_, dosing interval for the 2nd dose; τ_2_, dosing interval for the 3rd dose; τ_3_, dosing interval for the 4th dose; *t*_1_, infusion time in the LRRI phase; *t*_2_, infusion time in the LRCI phase; *t*_3_, or *t*_4_, duration in the elimination phase; ➀, AUC in the LRRI phase; ➁, AUC in the LRCI phase.

Drawing on our previous results on meropenem against highly resistant bacteria (Song et al., 2019) and the hypothesis stated by Pea et al. regarding the usefulness of continuous infusion for the treatment of borderline-susceptible pathogens (Pea and Viale, 2008), joint infusion mode with loading-rate rapid infusion (LRRI) and low-rate continuous infusion (LRCI) was speculated to be optimal and capable of yielding sufficient drug exposure for isolates with high MICs. Thus, the novel dosing strategy for VAN in this study represents an optimal two-step infusion (OTSI) mode, in which a portion of the VAN daily dose (*D*_*van*_) was administered at an approved maximum rate of 10 mg/min (i.e., 600 mg/h) within *t*_1_ in the LRRI phase to rapidly reach (but not limit) a trough serum concentration (C_*trough*_) of 15–20 mg/L (herein set as 20 mg/L). This phase was immediately followed by administration of the remainder of the *D*_*van*_ via LRCI within *t*_2_ to smoothly increase the concentration and maximize the daily area under the concentration-time curve (AUC_0__–__24_), as illustrated in Figure 1. To reduce VAN accumulation, in the OTSI design, the latter dose should be administered when the concentration from the previous dose falls to the *C*_*trough*_ value.

### Study Design

The PK/PD exposure, which is often considered an indicator of whether the infection is controllable or uncontrollable with antibacterial agents, of VAN was evaluated by Monte Carlo simulations (MCSs) and used to estimate the efficacy of VAN. The VAN-specific serum PK parameters [mainly the VAN clearance (*CL*_*van*_) and distribution volume (*V*_*d*_)] and microbiological susceptibility data for the targeted pathogens, together with the dosing parameters (mainly the dose, infusion duration, and rate), were incorporated into the mathematical model of the PK/PD index. MCSs were used to calculate the probabilities of target attainment (PTAs) at different MICs and the cumulative fractions of response (CFRs) for the targeted bacterial population with a pooled MIC distribution provided by each dosage regimen against the targeted bacterial species with doubling MICs between 0.125 and 64 mg/L for a given PK/PD target. A PTA or CFR of ≥90% and the causal dosage regimens were considered optimal.

### VAN Dosage Regimens

In general, each dose of VAN does not exceed 2 g, and the daily dose does not exceed 4 g given its dose-dependent nephrotoxicity (del Mar Fernández de Gatta Garcia et al., 2007; Lodise et al., 2008; USP, 2018), even for continuous infusion (Rybak et al., 2020b). However, to predict the interest of increasing the daily doses, simulations for higher doses (up to 6 g/day) were performed (del Mar Fernández de Gatta Garcia et al., 2007). In this study, 15 OTSI and 10 traditional intermittent i.v. infusion (TIII) regimens with daily doses of 2 g to up to 6 g for VAN were simulated, and the administration details for OTSI dosage regimens are shown in Table 1.

**TABLE 1:** Simulated dosage regimens and administration details.

**Models**	**TIII dosage regimens^a^**	**Administration details for OTSI dosage regimens**						
		**OTSI dosage regimens^a^**	**Volume of infusion solution (mL) ^b^**	**LRRI for VAN**		**LRCI for VAN**		**Infusion operation (by microcomputer pumping)**
				***v*_1_ (mg/h)**	***t*_1_ (h)**	***v*_2_ (mg/h)**	***t*_2_ (h)**	
2 g/day	0.5 g q 6 h or 1 g q 12 h	1.2 g LRRI + 0.8 g LRCI	500	600	2	36.36	22	2.5 mL/min (2 h) followed by 0.15 mL/min (22 h)
		1.8 g LRRI + 0.2 g LRCI	500	600	3	9.52	21	2.5 mL/min (3 h) followed by 0.04 mL/min (21 h)
		1.95 g LRRI + 0.05 g LRCI	500	600	3.25	2.41	20.75	2.5 mL/min (3.25 h) followed by 0.01 mL/min (20.75 h)
3 g/day	1 g q 8 h or 1.5 g q 12 h	1.2 g LRRI + 1.8 g LRCI	750	600	2	81.82	22	2.5 mL/min (2 h) followed by 0.34 mL/min (22 h)
		1.8 g LRRI + 1.2 g LRCI	750	600	3	57.14	21	2.5 mL/min (3 h) followed by 0.24 mL/min (21 h)
		2.0 g LRRI + 1.0 g LRCI	750	600	3.33	48.48	20.67	2.5 mL/min (3.33 h) followed by 0.20 mL/min (20.67 h)
4 g/day	1 g q 6 h or 2 g q 12 h	1.2 g LRRI + 2.8 g LRCI	1,000	600	2	127.27	22	2.5 mL/min (2 h) followed by 0.53 mL/min (22 h)
		1.8 g LRRI + 2.2 g LRCI	1,000	600	3	104.76	21	2.5 mL/min (3 h) followed by 0.44 mL/min (21 h)
		2.0 g LRRI + 2.0 g LRCI	1,000	600	3.33	96.86	20.67	2.5 mL/min (3.33 h) followed by 0.40 mL/min (20.67 h)
5 g/day	1.25 g q 6 h or 1.67 g q 8 h	1.2 g LRRI + 3.8 g LRCI	1,250	600	2	172.73	22	2.5 mL/min (2 h) followed by 0.72 mL/min (22 h)
		1.8 g LRRI + 3.2 g LRCI	1,250	600	3	152.38	21	2.5 mL/min (3 h) followed by 0.63 mL/min (21 h)
		2.0 g LRRI + 3.0 g LRCI	1,250	600	3.33	145.14	20.67	2.5 mL/min (3.33 h) followed by 0.60 mL/min (20.67 h)
6 g/day	1.5 g q 6 h or 2 g q 8 h	1.2 g LRRI + 4.8 g LRCI	1,500	600	2	218.18	22	2.5 mL/min (2 h) followed by 0.91 mL/min (22 h)
		1.8 g LRRI + 4.2 g LRCI	1,500	600	3	200.00	21	2.5 mL/min (3 h) followed by 0.83 mL/min (21 h)
		2.0 g LRRI + 4.0 g LRCI	1,500	600	3.33	193.52	20.67	2.5 mL/min (3.33 h) followed by 0.81 mL/min (20.67 h)

*^*a*^ according to the OTSI design, at the targeted *C*_*t*__*rough*_ of 20 mg/L and maximum limit of 2 g per dose (Lodise et al., 2008; Filippone et al., 2017), the dose of VAN administered in the LRRI phase ranges from 1.2 g (i.e., the minimum dose for the targeted *C*_*t*__*rough*_ according to the equation of *C*_*t*__*rough*_) to 2 g (i.e., the maximum limit of per dose), ^*b*^ determined by the final concentration of 2.5–5 g/L for VAN (USP, 2018). *t*_1_, infusion time in the LRRI phase; *t*_2_, infusion time in the LRCI phase; *v*_1_, zero-order infusion rate in the LRRI phase; *v*_2_, zero-order infusion rate in the LRCI phase.*

### Reoptimization of the PK/PD Target and Calculation of the PK/PD Index

The 2020 VAN guidelines recommend a target AUC_0__–__24_/MIC of 400–600 for a VAN MIC against MRSA of ≤1 mg/L (Rybak et al., 2020b). Herein, it should be noted that the upper limit of the AUC_0__–__24_/MIC target of 600, associated with nephrotoxicity, depends on an assumptive MIC of 1 mg/L and an AUC_0__–__24_ of 600 as determined by the daily dose based on the formula AUC_0__–__24_ = *D*_*van*_/*CL*_*van*_ (Rybak et al., 2020a). For an MIC of <1 mg/L, a daily dose with an AUC_0__–__24_ of 600 may show an AUC_0__–__24_/MIC of >600 and still reduced nephrotoxicity, implying that an AUC_0__–__24_/MIC of >600 for a specific MIC at the permissible daily dose is acceptable due to high variability among MICs between strains. Although a target AUC_0__–__24_/MIC of 400–600 is currently recommended as the primary PK/PD predictor for the treatment of serious MRSA infections, given that a VAN trough concentration of above 20 mg/L may be associated with increased risk of nephrotoxicity, a target AUC_0__–__24_/MIC of ≥400 (with no upper limit at a permissible daily dose) based on a target *C*_*trough*_ of 15–20 mg/L (herein set as 20 mg/L) might be an ideal and reliable PK/PD predictor and was thus used as the optimal index in this study, as recommended by Kullar et al. (2011).

Of note, the AUC_0__–__24_/MIC of ≥400 value used as an optimal target in this study might be more suitable for MRSA bloodstream infections (Rybak et al., 2020b). Therefore, for infections located at various sites, this value must be corrected based on the VAN tissue permeability since VAN penetration into infected tissues or fluids, to provide pharmacologically active drug concentrations at the site of action, is critical for predicting therapeutic responses. Data on VAN tissue permeability was obtained from previously published studies on adult patients (preferably those describing infection studies when available) with normal renal function [i.e., creatinine clearance (*CL*_*cr*_) ≥70 ml/min] or healthy volunteers (when the desired data from infected populations were unavailable), including those undergoing various surgeries. Table 2 is a collection of data on VAN penetration coefficient into some common tissues (Massias et al., 1992; Cruciani et al., 1996; Albanèse et al., 2000; Kitzes-Cohen et al., 2000; Luzzati et al., 2000; Byl et al., 2003; Lodise et al., 2011). Considering the profiles of VAN tissue penetration, a corrected approximate value of AUC_0__–__24_/MIC of ≥1,000 for pneumonia and meningitis, AUC_0__–__24_/MIC of ≥667 for sternal osteomyelitis, AUC_0__–__24_/MIC of ≥400 for mastitis, pleuritis and bacteremia, and AUC_0__–__24_/MIC of ≥250 for bacterial pericarditis would indicate sufficient PK/PD exposure. Thus, these targets based on the target C_*trough*_ of 20 mg/L, were used to assess the PK/PD exposure of VAN for these infections. According to the previous version of the 2020 VAN guidelines issued in 2009 (called the 2009 VAN guidelines) (Rybak et al., 2009), the total AUC_0__–__24_/MIC and the free VAN AUC_0__–__24_/MIC (i.e., AUC_0__–__24_ × 50% protein binding/MIC) are interchangeably reported for VAN; thus, the AUC_0__–__24_/MIC calculation refers to the total AUC_0__–__24_/MIC in this study, and the formulas are as follows:

**TABLE 2:** VAN for some common infections and the corresponding tissue penetration coefficient.

**Infections**	**Corresponding infected tissues**	**VAN tissues penetration coefficient (i.e., ratio of the mean tissue/concomitant serum concentration or AUC)**	**References**
Pneumonia	Lung or epithelial lining fluid	0.41	Cruciani et al. (1996), Lodise et al. (2011)
Meningitis	Cerebrospinal fluid	0.39	Albanèse et al. (2000)
Sternal osteomyelitis	Sternal bones	0.57	Massias et al. (1992)
Mastitis	Capsular tissue	1.06	Luzzati et al. (2000)
Bacterial pericarditis	Pericardium	1.6	Kitzes-Cohen et al. (2000)
Pleuritis	Pleural fluid	0.86	Byl et al. (2003)
Bacteremia	Bloodstream	1	NN

*NN, not necessary.*

(i) Formulas for OTSI (Eq. 1 and Eq. 2) (see Appendix: derivation of equations):

(1) $$C_{\text{trough}}=\frac{v_{1}}{C\,L_{\text{van}}}\cdot \left(1-e^{-C\,L_{\text{van}}\,/\,V_{\text{d}}\cdot t_{1}}\right)$$

$${\text{AUC}}_{\text{0-24}}/\text{MIC}=$$

$$\frac{\begin{array}{c} e^{-\frac{C\,L_{v\,a\,n}}{V_{d}}\,\left(t_{2}-t_{1}\right)}\{t_{2}\cdot e^{\frac{C\,L_{v\,a\,n}}{V_{d}}\cdot t_{2}}[CL_{v\,a\,n}\cdot v_{2}\cdot e^{\frac{C\,L_{v\,a\,n}}{V_{d}}\cdot t_{1}}\\ +CL_{v\,a\,n}\cdot v_{1}\left(e^{\frac{C\,L_{v\,a\,n}}{V_{d}}\cdot t_{1}}-1\right)+C_{t\,r\,o\,u\,g\,h}\cdot CL_{v\,a\,n}^{2}\cdot e^{\frac{C\,L_{v\,a\,n}}{V_{d}}\cdot t_{1}}]\\ +V_{d}\cdot v_{2}\cdot e^{\frac{C\,L_{v\,a\,n}}{V_{d}}\cdot t_{1}}\}\\ +e^{-\frac{C\,L_{v\,a\,n}}{V_{d}}\cdot t_{1}}[t_{1}\cdot e^{\frac{C\,L_{v\,a\,n}}{V_{d}}\cdot t_{1}}\left(CL_{v\,a\,n}\cdot v_{1}+C_{t\,r\,o\,u\,g\,h}\cdot CL_{v\,a\,n}^{2}\right)\\ +V_{d}\cdot v_{1}] \end{array}}{\text{MIC}\cdot C\,L_{v\,a\,n}^{2}}$$

(2) $$\,-\frac{V_{d}\,\left(v_{2}+v_{1}\right)}{\text{MIC}\cdot C\,L_{v\,a\,n}^{2}}$$

(ii) Formula for TIII (Eq. 3):

(3) AUC/0-24MIC=D/van(CL⋅vanMIC)

Eq. 3 was modified from the AUC_0__–__24_ formula derived from Moise-Broder et al. (2004a) as follows: AUC_0__–__24_ = dose per 24 h/[(α × *CL*_*cr*_ + β) × γ] [i.e., AUC_0__–__24_ = *D*_*van*_/*CL*_*van*_ since the relationship of *CL*_*van*_ and *CL*_*cr*_ is linear in patients with various degrees of impaired renal function (Rodvold et al., 1988)],

where C_*trough*_ (mg/L) is the targeted trough serum concentration; AUC_0__–__24_ (mg⋅h/L) is the daily area under the concentration-time curve; MIC (mg/L) is the minimum inhibitory concentration; *CL*_*van*_ (L/h) is the VAN clearance; *V*_*d*_ (L) is the distribution volume at the steady state; *t*_1_ (h) is the infusion time in the LRRI phase; *t*_2_ (h) is the infusion time in the LRCI phase; *v*_1_ (mg/h) is the zero-order infusion rate of 10 mg/min (i.e., 600 mg/h) in the LRRI phase; *v*_2_ (mg/h) is the zero-order infusion rate in the LRCI phase, which is calculated as the dose in the LRCI phase divided by the infusion time [i.e., (*D*_*van*_−600 × *t*_1_)/*t*_2_)]; ∫ is the integral operator; *dt* is the differential operator; *e* is the natural constant; *ln* is the natural logarithm; and α, β, γ are constants.

### Key VAN Population PK Parameters

Previous studies have established various models for the key population PK parameters (mainly *CL*_*van*_ and *V*_*d*_) for VAN among adult populations. However, some large cohort studies paid more attention to critically ill patients (Llopis-Salvia and Jiménez-Torres, 2006; Revilla et al., 2010; Roberts et al., 2011; Udy et al., 2013a; Mangin et al., 2014; Medellín-Garibay et al., 2017), as they are likely to show more PK variability than other populations due to their markedly varying physiological statuses (Roberts et al., 2014). However, PK changes to the antimicrobial volume of distribution, clearance, protein binding and tissue penetration in critically ill patients can be significantly different from those in other patient groups (Udy et al., 2013b), often resulting in insufficient antimicrobial concentrations in plasma and at the site of infection relative to those in the general population. Generally, accepted PK/PD targets may not be applicable for these patients (Roberts et al., 2014), potentially because it is more acceptable for these models to be used in critically ill patients. The population PK parameter models based on systemically infected patients from the study conducted by Matzke et al. (1984) [i.e., *CL*_*van*_ (ml/min) = 3.66 + 0.689 × *CL*_*cr*_ (ml/min) and *V*_*d*_ (L) = 0.72 L/kg (if *CL*_*cr*_ > 60 ml/min) or *V*_*d*_ (L) = 0.89 L/kg (if 10 ml/min ≤ *CL*_*cr*_ ≤ 60 ml/min) or *V*_*d*_ (L) = 0.9 L/kg (if *CL*_*cr*_ < 10 ml/min)] were used for our analysis since these models can be utilized to devise dosing schedules for patients with any degree of renal impairment and with normal renal function (Matzke et al., 1984) and because they performed well with satisfactory precision and bias prediction errors among eleven models in an external validation evaluation (Sánchez et al., 2010). Herein, this study focused on only adult patients with normal renal function (i.e., *CL*_*cr*_ > 70 ml/min), and the estimations of *CL*_*van*_ of (3.84 ± 1.14) L/h and *V*_*d*_ of (48.82 ± 3.74) L based on the demographic characteristics of the subjects in the Matzke et al. study (Matzke et al., 1984) were used to predict the efficacy of VAN.

### Microbiological Susceptibility Data

Based on the indications of VAN (USP, 2018) and the Antimicrobial Testing Leadership and Surveillance (ATLAS) database (The Micron Group, 2018), the targeted bacterial species with BS_*H–VAN–M*_, including mainly *E. faecalis, E. faecium, S. aureus, S. epidermidis*, and *S. bovis*, were modeled to determine the competence of VAN for BS_*H–VAN–M*_. The susceptibility data of these species, including the isolate numbers and the corresponding MIC frequency distributions, were derived from the ATLAS database between 2004 and 2018 (The Micron Group, 2018) and are displayed in Table 3.

**TABLE 3:** MIC frequency distributions of isolates for the targeted bacterial species.

**Bacterial species**	**Total (No.)**	**MIC (mg/L) frequency distributions [no. of the isolates (% of no.)]**					
		**≤0.0625**	**0.125**	**0.25**	**0.5**	**1**	**2**
*E. faecalis*	26058	0(0.00)	61(0.23)	186 (0.71)	2854 (10.95)	15104 (57.96)	6734 (25.84)
*E. faecium*	12923	0(0.00)	35(0.27)	294 (2.28)	3646 (28.21)	3987 (30.85)	604 (4.67)
*S. aureus*	120172	0(0.00)	193(0.16)	983 (0.82)	36605 (30.46)	74052 (61.62)	8332 (6.93)
*S. epidermidis*	9641	0(0.00)	8(0.08)	25 (0.26)	140 (1.45)	3941 (40.88)	5405 (56.06)
*S. bovis*	39	0(0.00)	1(2.56)	19 (48.72)	15 (38.46)	3 (7.69)	1 (2.56)

		**4**	**8**	**16**	**32**	**64**	**≥128**

*E. faecalis*	26058	427(1.64)	143(0.55)	48 (0.18)	55 (0.21)	446 (1.71)	0 (0.00)
*E. faecium*	12923	171(1.32)	162(1.25)	97 (0.75)	379 (2.93)	3548 (27.45)	0 (0.00)
*S. aureus*	120172	7(0.01)	0(0.00)	0 (0.00)	0 (0.00)	0 (0.00)	0 (0.00)
*S. epidermidis*	9641	115(1.19)	7(0.07)	0 (0.00)	0 (0.00)	0 (0.00)	0 (0.00)
*S. bovis*	39	0(0.00)	0(0.00)	0 (0.00)	0 (0.00)	0 (0.00)	0 (0.00)

### MCSs (Evaluation of Dosage Schedules)

Oracle Crystal Ball (version 11.1.2; Decisioneering Inc., Denver, CO, United States), a leading spreadsheet (i.e., Excel)-based application for predictive modeling, forecasting, simulation, and optimization, was used to perform the MCSs to calculate the probability of each dosing regimen to achieve the given combined PK/PD target against isolates with MICs of 0.125, 0.25, 0.5, 1, 2, 4, 8, 16, 32, and 64 mg/L, which is referred to as the PTA. This method has been well described elsewhere (Moine et al., 2016; Song and Long, 2018). In general, MCSs includes the following five steps: (1) data inputting (i.e., inputting the simulation variables and their representative values into the Excel table cells), (2) distribution pattern settings of simulation variables (i.e., setting the distribution patterns of the simulation variables according to their characteristics), (3) predictive variable settings and calculation (i.e., setting the variable that can reflect the drug efficacy as the predictive variable and calculating its typical value based on the mathematical model of it established on the simulation variables), (4) simulation parameter settings and execution (i.e., setting the number of simulations and the confidence interval), and (5) simulation result analysis (including the sensitivity and trend analysis, report creation and extraction of data). Briefly, in this study, assumed lognormal distributions of *CL*_*van*_ (3.84 ± 1.14 L/h) and *V*_*d*_ (48.82 ± 3.74 L); custom distributions of MIC frequency distributions (e.g., for *E. faecalis*, MIC = 0.125, 0.25, 0.5, 1, 2, 4, 8, 16, 32, and 64 mg/L, probability = 0.23%, 0.71%, 10.95%, 57.96%, 25.84%, 1.64%, 0.55%, 0.18%, 0.21%, and 1.71%, respectively); C_*trough*_ (constant given values = 20 mg/L, probability = 100%); and the infusion time and infusion rate and dose (e.g., for the regimen of 1.2 g LRRI + 0.8 g LRCI, *t*_1_ = 2 h, probability = 100%; *t*_2_ = 22 h, probability = 100%; *v*_1_ = 600 mg/h, probability = 100%; *v*_2_ = 36.36 mg/h, probability = 100%; dose in LRRI phase = 1200 mg, probability = 100%; *D*_*van*_ = 2000 mg, probability = 100%) were used as the simulation variables incorporated into the mathematical model of the predictive variables (i.e., C_*trough*_ and AUC_0__–__24_/MIC). The confidence interval was set to 95%. A 5,000-subject MCSs was performed on the predictive variable to obtain the PTA-predictive variable diagram, with the predictive variable as the abscissa and the PTA as the ordinate. The PTA at a given threshold of the predictive variable was obtained by specifying the abscissa as the given threshold. The overall expected value for the PTA (i.e., CFR) is related to PD target attainment in that it expresses the probability of a given dosage regimen achieving the desired exposures against an entire population of pathogens. Calculation of the CFR (Eq. 4) for each organism was performed by multiplying the PTA at each MIC by the percentage of isolates of each of the modeled organisms actually found at that MIC. A regimen with a PTA or CFR of ≥90% for the given PK/PD target was considered optimal.

(4) Eq.4:CFR=ΣPTA×iFi

where PTA_*i*_ is the probability of target attainment at a specific MIC value and F_*i*_ is the fraction of the targeted isolate actually found at that MIC in populations.

## Results

### PTAs for the Targeted C_trough_ Values

**FIGURE 2:**
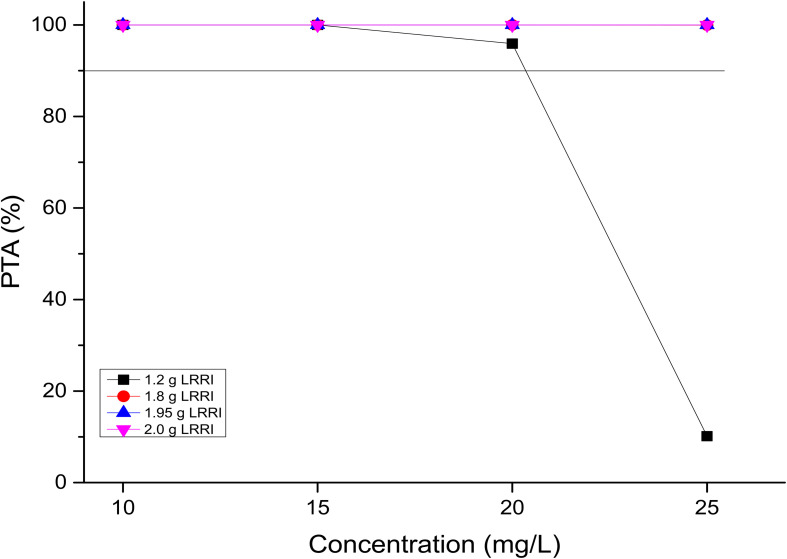
PTAs for various *C*_*trough*_ targets. According to the OTSI design (see Figure 1), the *C*_*trough*_ target is determined by the dose administered in the LRRI phase for each OTSI regimen.

PTAs versus the concentration profiles for simulations of the 15 OTSI regimens are presented in Figure 2. All of them achieved a PTA of ≥90% for the targeted C_*trough*_ of 20 mg/L, indicating that all regimens attained the predetermined C_*trough*_ target and thus laid the foundation to achieve the AUC_0__–__24_/MIC target established for this targeted C_*trough*_.

### PTAs for the Targeted AUC_0__–__24_/MIC Values

**FIGURE 3:**
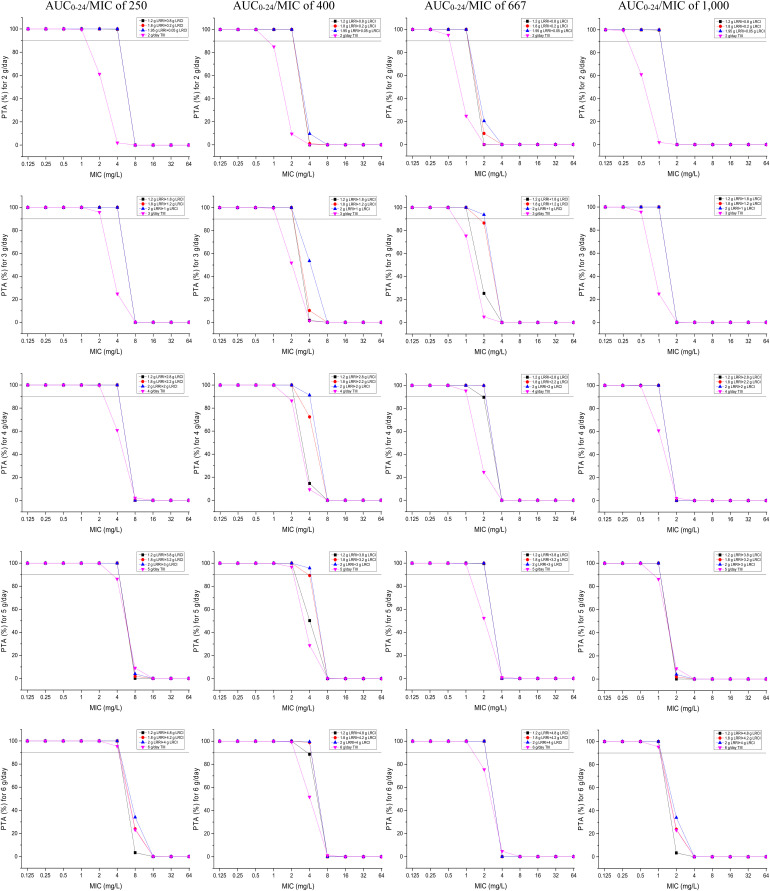
PTAs for various AUC_0–24_/MIC targets for MICs of up to 64 mg/L. Considering the profiles of VAN tissue penetration, simulating the target AUC_0–24_/MIC of 1,000 is for predicting VAN exposure in lung interstitial fluid and cerebrospinal fluid (i.e., for pneumonia and meningitis), 667 is for predicting VAN exposure in sternal bones (i.e., for sternal osteomyelitis), 400 is for predicting VAN exposure in capsular tissue, pleural fluid, and bloodstream (i.e., for mastitis, pleuritis, and bacteremia), and 250 is for predicting VAN exposure in pericardium (i.e., for bacterial pericarditis).

PTAs versus the various AUC_0__–__24_/MIC targets for simulations of the tested regimens are displayed in Figure 3. All of the OTSI regimens and the TIII regimens with a daily dose of up to 6 g for the AUC_0__–__24_/MIC target of 250 achieved a PTA of ≥90% at an MIC of up to 4 mg/L, while only the OTSI regimens with a daily dose of ≥4 g (e.g., 2 g LRRI + 2 g LRCI, 2 g LRRI + 3 g LRCI, 2 g LRRI + 4 g LRCI) for the AUC_0__–__24_/MIC target of 400 achieved this PTA result for an MIC of up to 4 mg/L, indicating that VAN at 4 g/day can generate a sufficient PK/PD response for MICs of up to 4 mg/L at the AUC_0__–__24_/MIC target of 400 when administered via OTSI for this dose. Surprisingly, 2 g/day VAN at the regimen of 1.95 g LRRI + 0.05 g LRCI still reached a PTA of ≥90% at an MIC of 2 mg/L. However, only OTSI regimens with a daily dose of ≥3 g obtained the optimal PTA for an MIC of up to 2 mg/L at an AUC_0__–__24_/MIC of 667, but only a MIC of up to 1 mg/L at an AUC_0__–__24_/MIC of 1,000 of one independent regimen was applied. As expected, with the same daily dose, the OTSI regimens displayed superior PK/PD exposure relative to the TIII regimens regardless of the PD targets. Table 4 summarizes the coverages of the tested regimens for the targeted bacterial isolate with different MICs at the condition of achieving ≥90% PTA.

**TABLE 4:** Coverage of the regimens for the isolates and/or for the targeted pathogen populations.

**Models**	**Dosage regimen**	**Covered pathogen isolates and/or populations in various types of the infection at different PD targets**			
		**AUC_0__–__24_/MIC of 250**	**AUC_0__–__24_/MIC of 400**	**AUC_0__–__24_/MIC of 667**	**AUC_0__–__24_/MIC of 1,000**
		
		**Mainly for bacterial pericarditis**	**Mainly for mastitis, pleuritis, and bacteremia**	**Mainly for sternal osteomyelitis**	**Mainly for pneumonia and meningitis**
2 g/day	1.2 g LRRI + 0.8 g LRCI	P_4_ + EFS, SA, SE, SB	P_2_ + EFS, SA, SE, SB	P_1_ + SA, SB	P_1_ + SA, SB
	1.8 g LRRI + 0.2 g LRCI	P_4_ + EFS, SA, SE, SB	P_2_ + EFS, SA, SE, SB	P_1_ + SA, SB	P_1_ + SA, SB
	1.95 g LRRI + 0.05 g LRCI	P_4_ + EFS, SA, SE, SB	P_2_ + EFS, SA, SE, SB	P_1_ + SA, SB	P_1_ + SA, SB
	2 g TIII	P_1_ + SA, SB	P_0_._5_ + SB	P_0_._5_	P_0_._25_
3 g/day	1.2 g LRRI + 1.8 g LRCI	P_4_ + EFS, SA, SE, SB	P_2_ + EFS, SA, SE, SB	P_1_ + SA, SB	P_1_ + SA, SB
	1.8 g LRRI + 1.2 g LRCI	P_4_ + EFS, SA, SE, SB	P_2_ + EFS, SA, SE, SB	P_1_ + EFS, SA, SE, SB	P_1_ + SA, SB
	2.0 g LRRI + 1.0 g LRCI	P_4_ + EFS, SA, SE, SB	P_2_ + EFS, SA, SE, SB	P_1_ + EFS, SA, SE, SB	P_1_ + SA, SB
	3 g TIII	P_2_ + EFS, SA, SE, SB	P_1_ + SB	P_0_._5_ + SB	P_0_._5_ + SB
4 g/day	1.2 g LRRI + 2.8 g LRCI	P_4_ + EFS, SA, SE, SB	P_2_ + EFS, SA, SE, SB	P_1_ + EFS, SA, SE, SB	P_1_ + SA, SB
	1.8 g LRRI + 2.2 g LRCI	P_4_ + EFS, SA, SE, SB	P_2_ + EFS, SA, SE, SB	P_2_ + EFS, SA, SE, SB	P_1_ + SA, SB
	2.0 g LRRI + 2.0 g LRCI	P_4_ + EFS, SA, SE, SB	P_4_ + EFS, SA, SE, SB	P_2_ + EFS, SA, SE, SB	P_1_ + SA, SB
	4 g TIII	P_2_ + EFS, SA, SE, SB	P_1_ + EFS, SA, SE, SB	P_1_ + SA, SB	P_0_._5_ + SB
5 g/day	1.2 g LRRI + 3.8 g LRCI	P_4_ + EFS, SA, SE, SB	P_2_ + EFS, SA, SE, SB	P_2_ + EFS, SA, SE, SB	P_1_ + SA, SB
	1.8 g LRRI + 3.2 g LRCI	P_4_ + EFS, SA, SE, SB	P_2_ + EFS, SA, SE, SB	P_2_ + EFS, SA, SE, SB	P_1_ + SA, SB
	2.0 g LRRI + 3.0 g LRCI	P_4_ + EFS, SA, SE, SB	P_4_ + EFS, SA, SE, SB	P_2_ + EFS, SA, SE, SB	P_1_ + SA, SB
	5 g TIII	P_2_ + EFS, SA, SE, SB	P_2_ + EFS, SA, SE, SB	P_1_ + SA, SB	P_0_._5_ + SB
6 g/day	1.2 g LRRI + 4.8 g LRCI	P_4_ + EFS, SA, SE, SB	P_2_ + EFS, SA, SE, SB	P_2_ + EFS, SA, SE, SB	P_1_ + SA, SB
	1.8 g LRRI + 4.2 g LRCI	P_4_ + EFS, SA, SE, SB	P_4_ + EFS, SA, SE, SB	P_2_ + EFS, SA, SE, SB	P_1_ + SA, SB
	2.0 g LRRI + 4.0 g LRCI	P_4_ + EFS, SA, SE, SB	P_4_ + EFS, SA, SE, SB	P_2_ + EFS, SA, SE, SB	P_1_ + SA, SB
	6 g TIII	P_4_ + EFS, SA, SE, SB	P_2_ + EFS, SA, SE, SB	P_1_ + SA, SB	P_1_ + SA, SB

*EFS, *E. faecalis*; SA, *S. aureus*; SE, *S. epidermidis*; SB, *S. bovis*. P_0_._25_, P_0_._5_, P_1_, P_2_, and P_4_ signify the pathogen isolates with MICs of up to 0.25, 0.5, 1, 2, and 4 mg/L, respectively. Regimens with P_*x*_ (x = 0.25, 0.5, 1, 2, or 4) + y (y = EFS, SA, SE, SB, or a combination) signify that they are competent for the treatment of infections caused by the pathogen isolates actually found at that MIC if the exact MIC values are available and/or for the treatment of infections caused by the targeted pathogen populations identified with only bacterial species if the exact MIC values are unavailable.*

### CFRs for the Targeted Bacterial Species

**FIGURE 4:**
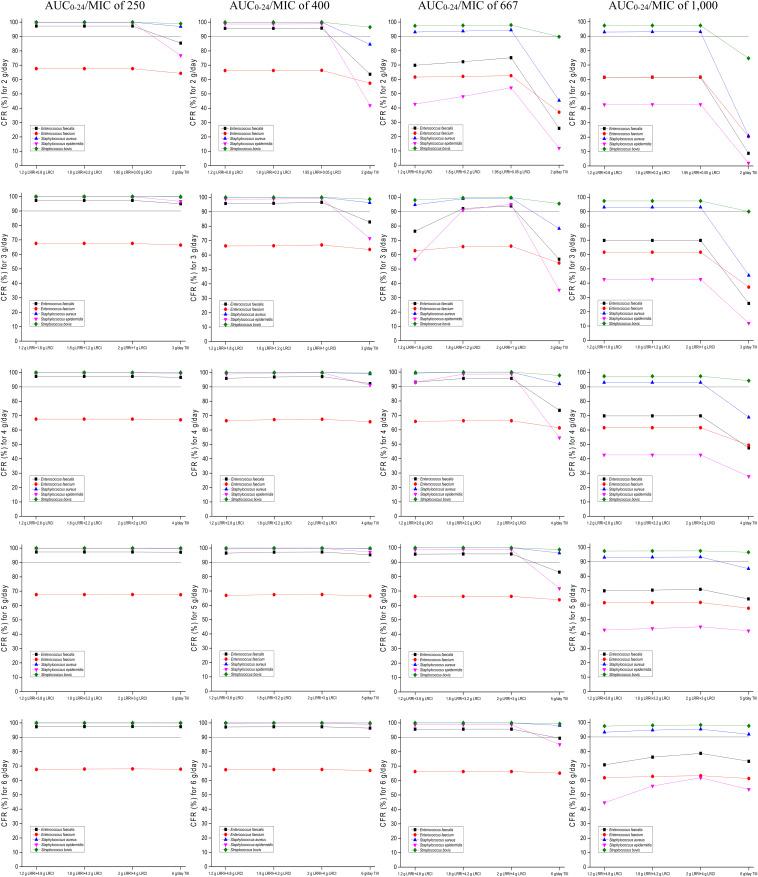
CFRs of achieving AUC_0–24_/MIC targets for the targeted bacterial species with pooled MIC distributions. Considering the profiles of VAN tissue penetration, simulating the target AUC_0–24_/MIC of 1,000 is for predicting VAN exposure in lung interstitial fluid and cerebrospinal fluid (i.e., for pneumonia and meningitis), 667 is for predicting VAN exposure in sternal bones (i.e., for sternal osteomyelitis), 400 is for predicting VAN exposure in capsular tissue, pleural fluid, and bloodstream (i.e., for mastitis, pleuritis, and bacteremia), and 250 is for predicting VAN exposure in pericardium (i.e., for bacterial pericarditis).

The CFRs versus various targeted bacterial species for simulations of the tested regimens are displayed in Figure 4. No regimens achieved a CFR of ≥90% for the *E. faecium* population regardless of the PD targets. Based on currently pooled MIC distributions and an AUC_0__–__24_/MIC of ≤400, the OTSI regimens, even at 2 g/day, yielded a CFR of ≥90% for the *E. faecalis, S. aureus, S. epidermidis*, and *S. bovis* populations. However, at an AUC_0__–__24_/MIC of 667, the OTSI regimens with a daily dose of ≥3 g for these pathogen populations achieved the requisite CFR, while those with a daily dose of 2 g achieved the requisite CFR for only the *S. aureus and S. bovis* populations. Unfortunately, all OTSI regimens covered only the *S. aureus and S. bovis* populations at an AUC_0__–__24_/MIC of 1,000. At the classical target of an AUC_0__–__24_/MIC of 400, the TIII regimens with only ≥4 g/day achieved a CFR of ≥90% for the *E. faecalis, S. aureus, S. epidermidis*, and *S. bovis* populations. Table 4 summarizes the coverages of the tested regimens for the targeted bacterial species with pooled MIC distributions at the condition of achieving ≥90% CFR.

## Discussion

To our knowledge, this study is the first to design OTSI method to exploit the maximum potentialities of VAN. Our data suggests great superiority and satisfactory PK/PD exposure of the dosing strategy of OTSI relative to that of TIII, especially for BS_*H–VAN–M*_ of ≥2 mg/L and ≤4 mg/L, supporting that VAN in OTSI mode is still powerful for BS_*H–VAN–M*_ of ≤4 mg/L and thus providing a temporary solution when better treatment options are unavailable.

Currently, it is still difficult to find an antimicrobial with a desired balance of safety, efficacy, availability, cost, and other potential issues for BS_*H–VAN–M*_ despite the emergence of some current novel drugs (Gould, 2013; Barber et al., 2015). Importantly, the lack of clinical trials and experimental studies evaluating antimicrobial efficacy against BS_*H–VAN–M*_ has forced clinicians to rely on alternative regimens extrapolated from PK/PD models, retrospective studies, and case reports (Yim et al., 2017). Currently, clinical data on the continued use of VAN in the setting of known BS_*H–VAN–M*_ are quite rare. Some clinical and simulated studies have focused mainly on MRSA isolates with an MIC of 2 mg/L (still within the susceptibility range).

Clinically, despite the achievement of a target C_*trough*_ of 15 mg/L, VAN performed worse against MRSA bacteremia, intraabdominal infections and pneumonia caused by isolates with an MIC of 2 mg/L than against those with an MIC < 2 mg/L (Moise-Broder et al., 2004b; Sakoulas et al., 2004; Hidayat et al., 2006). Harigaya et al. (2009) demonstrated that VAN did not have bactericidal activity against MRSA pneumonia due to infection by isolates with an MIC of 1 mg/L regardless of whether 1–1.5 g of VAN was administered twice daily (i.e., 2–3 g/day) and of the assumptions of 100% VAN penetration in the lung and an AUC_0__–__24_/MIC of 350 as the therapeutic target. Likewise, for MRSA meningitis due to strains with MICs of 2 mg/L, Sipahi et al. (2013) found that six (five of the six had MRSA isolates with a VAN MIC of 2 mg/L) out of eight patients (75%) with MRSA meningitis who received 500 mg VAN every 6 h (i.e., 2 g/day) as a 1 h infusion failed to achieve the clinical outcome, suggesting that VAN, at its standard daily dose and conventional dosing strategy, is unsatisfactory for such infections. Interestingly, our data indicated that even for bacteremia due to isolates with MICs of 2 mg/L, VAN at the standard 2 g/day can still produce adequate PK/PD exposure when administered using the regimen of 1.2 g LRRI + 0.8 g LRCI, 1.8 g LRRI + 0.2 g LRCI, or 1.95 g LRRI + 0.05 g LRCI, while worse outcomes were achieved when TIII was used; these results suggest the competence of VAN for such infections and the superiority of OTSI relative to TIII. Consistently, however, for pneumonia and meningitis due to isolates with MICs of 2 mg/L, VAN performed poorly (well only for those resulting from isolates with MICs of ≤1 mg/L) even at 6 g/day and with the OTSI strategy. The occurrence of this outcome may be due to the fact that VAN has poor profiles in terms of lung interstitial fluid and cerebrospinal fluid penetration. More recently, a review research for the penetration of VAN (15 mg/kg IV 1 h) into bone showed an average VAN concentration of 3.8 mg/L and 4.5 mg/L in cancellous and cortical bone, respectively (Thabit et al., 2019), suggesting that VAN reaches concentrations that exceed the MIC_90_ against *S. aureus* (1 mg/L) and thus implying that VAN will have good performance on bone infections due to such bacteria. However, in a case report, Lee et al. (2016) found that the infusion of even 1 g of VAN over 1 h every 12 h (i.e., 2 g/day) was unsuccessful for an infected patient who had lumbar osteomyelitis, discitis, and epidural abscess with persistent MRSA bacteremia resulted from isolates with an MIC of 1 mg/L. The occurrence of this result may be associated with the physiological and pathological status of the patient, slow bactericidal activity of VAN, inappropriate dosage regimen of VAN or a combination. However, our data showed that even though relatively poor bone penetration coefficient (0.57) of VAN was applied, 2 g/day VAN with a rational OTSI strategy (e.g., 1.2 g LRRI + 0.8 g LRCI) could still yield desirable PK/PD exposure for this complicated infection, and 4 g/day VAN with a rational OTSI regimen (e.g., 2 g LRRI + 2 g LRCI) performed well for this case due to isolates with an MIC of up to 2 mg/L. This implied that for population in which VAN has good profiles of bone penetration, these optimal dosage regimens will show better PK/PD exposure performance, and this was also the case for VAN against other infected tissues. Unexpectedly, for bacterial pericarditis, 2 g/day VAN with a rational OTSI (e.g., 1.2 g LRRI + 0.8 g LRCI) achieved optimal PK/PD exposure for isolates with an MIC of up to 4 mg/L, indicating good activity at infected sites with strong VAN penetration and enrichment (e.g., the pericardium).

Likewise, in the simulated studies, poor VAN exposure at an MIC of 2 mg/L was also observed with a PTA of 90% as an acceptable PK/PD exposure target. By MCSs, Mohr et al. (Mohr and Murray, 2007) determined the PTAs for the targeted AUC_0__–__24_/MIC of ≥400 using the PK data derived from the study conducted by Jeffres et al. (2006) and indicated that the PTA would be 100% at an MIC of 0.5 mg/L but 0% at an MIC of 2 mg/L despite that a high dose of VAN (i.e., C_*trough*_ > 15 mg/L but no detailed information on the simulated regimens) was used. Coincidentally, using MCSs, del Mar Fernández de Gatta Garcia et al. (2007) reported that 3–4 g/day VAN administered via the TIII strategy for an assumed MIC of 1 mg/L would reach a PTA of 90% at the targeted AUC_0__–__24_/MIC of 400, thus questioning the dosage of 2 g/day as a standard schedule for such isolates. Interestingly, Setiawan et al. (2019) found that 2 g/day VAN with TIII obtained a PTA of 100% at an MIC of 0.5 mg/L but only 84.41% at an MIC of 1 mg/L; moreover, even 4 g/day VAN administered with TIII failed to achieve the optimal PTA for an MIC of 2 mg/L. Conversely, however, our data supported that at the above PK/PD target, 2 g/day VAN, with the regimen of 1.2 g LRRI + 0.8 g LRCI, 1.8 g LRRI + 0.2 g LRCI, or 1.95 g LRRI + 0.05 g LRCI, still produced a PTA of ≥90% at an MIC of 2 mg/L, suggesting the achievement of sufficient PK/PD exposure. Surprisingly, 4 g/day VAN, with the regimen of 2.0 g LRRI + 2.0 g LRCI, yielded a PTA of ≥90% (91.28%) for an MIC of up to 4 mg/L but failed with the regimen of 4 g/day administered with TIII. In addition, when administered via TIII and even at a high dose of 5 g/day, VAN cannot provide sufficient PK/PD exposure for *Enterococcus* isolates with MICs of 4 mg/L regardless of the infection, although these isolates are currently considered to be susceptible to VAN at this breakpoint. These findings show the superiority and feasibility of OTSI for the competence mining of VAN against BS_*H–VAN–M*_. Of note, the achievement of such regimens for these MICs might be more agreeable for bacteremia, mastitis and pleuritis based on VAN tissue permeability and the derivation of the AUC_0__–__24_/MIC target of 400 [derived mainly from MRSA bloodstream infections (Rybak et al., 2020b)].

However, the exact MIC values are often unavailable in empirical therapy. Fortunately, our data provided the CFRs for the targeted bacterial populations. In empirical therapy, 3–4 g/day VAN with an OTSI strategy, such as the preferred regimen of 2 g LRRI + 1 g LRCI or 2 g LRRI + 2 g LRCI, may be preferable for most common infections, including bacterial pericarditis, mastitis, pleuritis, bacteremia and sternal osteomyelitis, as these regimens produced a CFR of ≥90% for all of the targeted bacterial populations, including *E. faecalis*, *S. aureus*, *S. epidermidis*, and *S. bovis*, with the sole exception of *E. faecium*. However, for pneumonia and meningitis, even at the aggressive regimen of 6 g/day (2 g LRRI + 4 g LRCI), VAN was sufficient for infections due to only *S. aureus* and *S. bovis*; in theory, for such pathogen populations, the standard 2 g/day may result in similar PK/PD exposure and reduced nephrotoxicity relative to that at 6 g/day based on our data. Table 5 summarizes some preferred VAN regimens for common infections based on our analysis.

**TABLE 5:** Preferred VAN regimen recommendations for some common infections.

**Infections**	**Available for MIC ^a^**				**Unavailable for MIC (i.e., in empirical therapy) ^b^**
	**≤1 mg/L**	**>1 mg/L and ≤2 mg/L**	**>2 mg/L and ≤4 mg/L**	**>4 mg/L**	
Bacterial pericarditis	1.95 g LRRI + 0.05 g LRCI	1.95 g LRRI + 0.05 g LRCI	1.95 g LRRI + 0.05 g LRCI	NA	1.95 g LRRI + 0.05 g LRCI
Mastitis, pleuritis, and bacteremia	1.95 g LRRI + 0.05 g LRCI	1.95 g LRRI + 0.05 g LRCI	2.0 g LRRI + 2.0 g LRCI	NA	1.95 g LRRI + 0.05 g LRCI
Sternal osteomyelitis	1.95 g LRRI + 0.05 g LRCI	2.0 g LRRI + 2.0 g LRCI	NA	NA	2.0 g LRRI + 1.0 g LRCI
Pneumonia and meningitis	1.95 g LRRI + 0.05 g LRCI	NA	NA	NA	1.95 g LRRI + 0.05 g LRCI

*^*a*^ determined by such a regimen that has a lower daily dose and a PTA of ≥90%; ^*b*^ determined by such a regimen that has a lower daily dose, a wider coverage of bacterial species and a CFR of ≥90%. NA, (VAN) not applicable.*

Despite acquisition of target AUC_0__–__24_/MIC by OTSI method, we were unable to demonstrate a microbiological or clinical superiority of these recommended VAN OTSI regimens based on MCSs as little data are currently available, either *in vivo* or *in vitro* experiments. However, in a study of *in vitro* pharmacodynamic model and MCSs (Eguchi et al., 2010), experimental verification of the efficacy of optimized two-step infusion therapy (OTIT), for meropenem but not for VAN, was performed. Surprisingly, the *in vitro* bactericidal effect of the optimal meropenem OTIT regimens is consistent with the bactericidal effect predicted by their respective PTA of these regimens derived from MCSs, especially for *Pseudomonas aeruginosa* isolates with meropenem MIC of ≤4 mg/L, which partly indicated the feasibility of MCSs method in predicting the efficacy of the regimens although the research object of this study was meropenem rather than VAN. However, despite this significative case of MCSs prediction, the recommended VAN OTSI regimens based on MCSs cannot be considered certainly effective against the target bacteria given the difference in action profiles among antibiotics and in resistance mechanisms among bacteria. In addition, modification of VAN delivery in severe infections may not be sufficient, by itself, to change the clinical outcome for critically ill patients, and the bacterial status like bacterial biofilm widely formed among various bacteria were also a barrier to effective anti-infection.

This study has some limitations. First, the desired regimens acquired by MCSs were not validated by experimental outcomes, which somewhat limits their generalization. However, further studies on this topic will be summarized in our next study. Second, the AUC_0__–__24_/MIC target of >600 (e.g., 667 and 1,000) utilized in this study may be too high to increase VAN-induced nephrotoxicity. Regarding this, we need to correctly understand (1) the derivation of the value of 600, and (2) that these targets simulated herein are just used to simulate concentrations in sites other than the plasma (e.g., lung interstitial fluid and cerebrospinal fluid). Moreover, the dosing interval and a C_*trough*_ value of 20 mg/L designed for OTSI method ensure a theoretically low probability of drug accumulation; notwithstanding this optimal design, however, therapeutic monitoring of VAN should be recommended as a routine test, especially for patients with long medication time. Third, in consideration of the problem of VAN stability and low flow rate (especially in the LRCI phase), some experts consider that OTSI may not be implemented well clinically. However, the concern on the VAN stability is not a factor preventing the implementation of OTSI method since VAN displayed well chemical stability even during 24-h infusion (Masse et al., 2020). And, the low flow rate can be performed well with the recently developed microcomputer pumping method. Moreover, we can appropriately increase the total solvent volume of VAN to improve the velocity problem. Fourth, the PK models selected in this study may have been biased for assessing target attainment in different populations. Indeed, the inherent interindividual variability in the PK data contributes to the prediction bias. However, we believe that the use of these PK models together with PK/PD analysis based on MCSs still offers a definitive outcome to some extent since (1) the PK variability, which is often extensive, was considered by the lognormal distribution patterns settings of *CL*_*van*_ and *V*_*d*_ in MCSs, and (2) the PK models used in this study performed well with satisfactory precision and bias prediction errors when compared with some established models in the external validation evaluation (Sánchez et al., 2010). Finally, at the same PK/PD target, whether regimens with desirable PK/PD exposure to MRSA are also satisfactory against *Enterococcus* and whether regimens with acceptable PK/PD exposure for ordinary patients are also adequate for critically ill patients remains unknown, thereby limiting the extrapolation of optimal regimens to other BS_*H–VAN–M*_ with different resistance mechanisms and to critically ill patients with marked PK variability. Notwithstanding its limitations, the study provides some tentative options for BS_*H–VAN–M*_, especially in the absence of better alternatives. However, prospective validation of these limitations is desirable.

## Conclusion

When faced with the daily challenge of infections due to isolates with high MICs, we should try to reduce the gap between the available medical evidence for using VAN and the dearth of alternative options, some of which have not been sufficiently explored and/or whose efficacy in certain situations remains debatable. The question of whether VAN can continue to be used for isolates with MICs of ≥2 mg/L (this study supports VAN, at an allowable daily dose and with a reasonable OTSI, for isolates with an MIC of up to 4 mg/L) is still not obsolete, and this study provides a new resource for understanding how PK/PD modeling shapes the power of VAN to meet the growing challenges of BS_*H–VAN–M*_ infections. However, in the absence of control trials, the continued appraisal of VAN for use, along with the optimal dosage regimens in clinical experience, will provide additional important information on its utility against BS_*H–VAN–M*_.

## Data Availability Statement

The original contributions presented in the study are included in the article/supplementary material, further inquiries can be directed to the corresponding author.

## Author Contributions

XS performed the model simulation and wrote the manuscript. MZ, YW, and YP conceptualized and supervised the manuscript. All authors approved the final version of the manuscript.

## Conflict of Interest

The authors declare that the research was conducted in the absence of any commercial or financial relationships that could be construed as a potential conflict of interest.

## Appendix (Derivation of Equations)

The two basic PK equations via i.v. infusion in the one-compartment model [derived from the book (Basic Pharmacokinetics and Pharmacodynamics: An Integrated Textbook and Computer Simulations, 1 edition. Hoboken, NJ, United States: Wiley; 2011) edited by Rosenbaum SE] are as follows:

(i) During infusion

(1) $$C=\frac{v}{C\,L}\cdot \left(1-e^{-C\,L\,/\,V\cdot t}\right)\left(0\leq t\leq t_{\inf}\right)$$

*C*_*max*_ in every dose is achieved when *t* is equal to *t*_*inf*_.

(ii) After completion of the infusion

(2) $$C=C_{\text{max}}\cdot e^{-C\,L/V\cdot t^{\prime }}\;\left(0\leq t^{\prime }<\infty \right)$$

1. Derivation of *C*_*trough*_

According to Figure 1, whether *C* can reach *C*_*trough*_ after the first dose is determined by Eq. 3:

(3) $$C_{\text{trough}}=\frac{v_{1}}{C\,L}\cdot \left(1-e^{-C\,L\,/\,V\cdot t_{1}}\right)$$

2. Derivation of AUC_0__–__24_/MIC

According to the OTSI design, the change in concentration reaches a steady state from the second dose. Thus, the AUC_0__–__24_/MIC at a steady state could be determined by an AUC_0__–__24_/MIC produced from the second dose. In the second dose, due to the existence of a basal concentration of *C*_*trough*_,

(4) $$C_{\mathrm{LRRI}-2}=C_{\text{trough}}+\frac{v_{1}}{C\,L}\cdot \left(1-e^{-C\,L\,/\,V\cdot t}\right)\left(0\leq t\leq t_{1}\right)$$

(5) $$C_{\mathrm{LRCI}-2}=C_{\text{trough}}+\frac{v_{1}}{C\,L}\cdot \left(1-e^{-C\,L/V\cdot t_{1}}\right)+\frac{v_{2}}{C\,L}\cdot \left(1-e^{-C\,L/V\cdot t}\right)\;\left(0\leq t\leq t_{2}\right)$$

According to Figure 1,

(6) AUC=0-24AUC+1AUC2

Due to the mathematical integral relationship of the AUC calculation with concentrations or the trapezoidal rule for the summation of curve area,

(7) AUC=∫Cdt

According to Eqs. 4 and 5 as well as the definite integration of AUC_1_ and AUC_2_,

(8) $${\text{AUC}}_{1}=\int_{0}^{t_{1}}\left[C_{\text{trough}}+\frac{v_{1}}{C\,L}\cdot \left(1-e^{-C\,L/V\cdot t}\right)\right]\,dt$$

(9) $${\text{AUC}}_{2}=\int_{0}^{t_{2}}\left[C_{\text{trough}}+\frac{v_{1}}{C\,L}\cdot \left(1-e^{-C\,L\,/\,V\cdot t_{1}}\right)+\frac{v_{2}}{C\,L}\cdot \left(1-e^{-C\,L\,/\,V\cdot t}\right)\right]\,dt$$

By transformation between the mathematical functions based on the online integral calculator (https://www.integral-calculator.com/),

(10) $${\text{AUC}}_{1}=\frac{e^{-\frac{C\,L}{V}\cdot t_{1}}\,\left[t_{1}\cdot e^{\frac{C\,L}{V}\cdot t_{1}}\,\left(C\,L\cdot v_{1}+C_{t\,r\,o\,u\,g\,h}\cdot C\,L^{2}\right)+V\cdot v_{1}\right]}{C\,L^{2}}-\frac{V\cdot v_{1}}{C\,L^{2}}$$

(11) $${\text{AUC}}_{2}=\frac{e^{-\frac{C\,L}{V}\,\left(t_{2}-t_{1}\right)}\,\left\{t_{2}\cdot e^{\frac{C\,L}{V}\cdot t_{2}}\,\left[C\,L\cdot v_{2}\cdot e^{\frac{C\,L}{V}\cdot t_{1}}+C\,L\cdot v_{1}\,\left(e^{\frac{C\,L}{V}\cdot t_{1}}-1\right)+C_{t\,r\,o\,u\,g\,h}\cdot C\,L^{2}\cdot e^{\frac{C\,L}{V}\cdot t_{1}}\right]+V\cdot v_{2}\cdot e^{\frac{C\,L}{V}\cdot t_{1}}\right\}}{C\,L^{2}}-\frac{V\cdot v_{2}}{C\,L^{2}}$$

$${\text{AUC}}_{0-24}=\frac{\begin{array}{c} e^{-\frac{C\,L}{V}\,\left(t_{2}-t_{1}\right)}\,\left\{t_{2}\cdot e^{\frac{C\,L}{V}\cdot t_{2}}\,\left[C\,L\cdot v_{2}\cdot e^{\frac{C\,L}{V}\cdot t_{1}}+C\,L\cdot v_{1}\,\left(e^{\frac{C\,L}{V}\cdot t_{1}}-1\right)+C_{t\,r\,o\,u\,g\,h}\cdot C\,L^{2}\cdot e^{\frac{C\,L}{V}\cdot t_{1}}\right]+V\cdot v_{2}\cdot e^{\frac{C\,L}{V}\cdot t_{1}}\right\}\\ +e^{-\frac{C\,L}{V}\cdot t_{1}}\,\left[t_{1}\cdot e^{\frac{C\,L}{V}\cdot t_{1}}\,\left(C\,L\cdot v_{1}+C_{t\,r\,o\,u\,g\,h}\cdot C\,L^{2}\right)+V\cdot v_{1}\right] \end{array}}{C\,L^{2}}$$

(12) $$\,-\frac{V\,\left(v_{2}+v_{1}\right)}{C\,L^{2}}$$

Notably,

$${\text{AUC}}_{0-24}\,/\,\text{MIC}=\frac{\begin{array}{c} e^{-\frac{C\,L}{V}\,\left(t_{2}-t_{1}\right)}\,\left\{t_{2}\cdot e^{\frac{C\,L}{V}\cdot t_{2}}\,\left[C\,L\cdot v_{2}\cdot e^{\frac{C\,L}{V}\cdot t_{1}}+C\,L\cdot v_{1}\,\left(e^{\frac{C\,L}{V}\cdot t_{1}}-1\right)+C_{t\,r\,o\,u\,g\,h}\cdot C\,L^{2}\cdot e^{\frac{C\,L}{V}\cdot t_{1}}\right]+V\cdot v_{2}\cdot e^{\frac{C\,L}{V}\cdot t_{1}}\right\}\\ +e^{-\frac{C\,L}{V}\cdot t_{1}}\,\left[t_{1}\cdot e^{\frac{C\,L}{V}\cdot t_{1}}\,\left(C\,L\cdot v_{1}+C_{t\,r\,o\,u\,g\,h}\cdot C\,L^{2}\right)+V\cdot v_{1}\right] \end{array}}{\text{MIC}\cdot C\,L^{2}}$$

(13) $$-\frac{V\,\left(v_{2}+v_{1}\right)}{\text{MIC}\cdot C\,L^{2}}$$

Where *C* (mg/L) is the drug serum concentration; *C*_*max*_ (mg/L) is the peak concentration; *v* (mg/h) is the zero-order infusion rate; *e* is the natural constant; *t* (h) is the infusion time; *t*_*inf*_ (h) is the maximum of infusion time; *t*’ (h) is the duration after infusion completion; *t*_1_ (h) is the infusion time in the LRRI phase; *t*_2_ (h) is the infusion time in the LRCI phase; *C*_*LRRI–*__2_ (mg/L) is the drug serum concentration in the LRRI phase after the second dose; *C*_*LRCI–*__2_ (mg/L) is the drug serum concentration in the LRCI phase after the second dose; AUC_0__–__24_ (mg⋅h/L) is the daily area under the concentration-time curve; AUC (mg⋅h/L) is the area under the concentration-time curve; AUC_1_ (mg⋅h/L) is the area under the concentration-time curve in the LRRI phase; AUC_2_ (mg⋅h/L) is the area under the concentration-time curve in the LRCI phase; AUC_0__–__24_/MIC (h) is the daily area under the concentration-time curve to the minimum inhibitory concentration ratio; *v*_1_ (mg/h) is the zero-order infusion rate in the LRRI phase; *v*_2_ (mg/h) is the zero-order infusion rate in the LRCI phase, which is calculated as the dose in the LRCI phase divided by the infusion time [i.e., (*D*-*v*_1_ × *t*_1_)/*t*_2_]; ∫ is the integral operator; *dt* is the differential operator; and *ln* is the natural logarithm.
